# Specialist Rehabilitation Providers’ Experiences With an Online Self-Compassion Course: Reflexive Thematic Analysis

**DOI:** 10.2196/81706

**Published:** 2026-07-15

**Authors:** Eva Cohen, Kristina M Kokorelias, Dorothy Luong, McKyla McIntyre, Marina Bastawrous Wasilewski, Sander L Hitzig, Sarah Munce, Rosalie Steinberg, Anthony Feinstein, Mark T Bayley, Lawrence R Robinson, Carolyn Steele Gray, Robert Simpson

**Affiliations:** 1 Evaluative Clinical Sciences, Sunnybrook Research Institute, Sunnybrook Health Sciences Centre, Toronto, ON, Canada; 2 Department of Geriatric Medicine, Sinai Health System and University Health Network, Toronto, ON, Canada; 3 Department of Occupational Science & Occupational Therapy, Temerty Faculty of Medicine, University of Toronto, Toronto, ON, Canada; 4 Rehabilitation Sciences Institute, Temerty Faculty of Medicine, University of Toronto, 550 University AveToronto, Toronto, ON, M5G 2A2, Canada, 1 416-360-4000 ext 40047; 5 KITE Research Institute, University Health Network, Toronto, ON, Canada; 6 Bloorview Research Institute, Holland Bloorview Kids Rehabilitation Hospital, Toronto, ON, Canada; 7 Toronto Rehabilitation Institute, University Health Network, University of Toronto, Toronto, ON, Canada; 8 Institute of Health Policy, Management and Evaluation, Dalla Lana School of Public Health, University of Toronto, Toronto, ON, Canada; 9 Department of Medicine, University of British Columbia, Victoria, BC, Canada; 10 Lunenfeld-Tanenbaum Research Institute, Sinai Health System, Toronto, ON, Canada; 11 Institute of Health and Wellbeing, University of Glasgow, Glasgow, United Kingdom

**Keywords:** self-compassion, burnout (Professional), rehabilitation, qualitative research, health personnel

## Abstract

**Background:**

Rehabilitation health care providers (HCPs) report high levels of burnout. Self-compassion interventions have shown beneficial effects on HCP burnout, but they have never been explored in specialist rehabilitation settings where challenges may differ.

**Objective:**

This study aimed to explore the experiences of specialist rehabilitation HCPs with an online Self-Compassion for Healthcare Communities (SCHC) course aimed at providing tools to regulate emotional well-being, including burnout.

**Methods:**

Semistructured qualitative interviews (n=20) with specialist rehabilitation HCPs were used to explore experiences with the SCHC course. A reflexive thematic analysis study design was chosen to highlight participants’ insights, and inductive coding was undertaken to analyze data and organize findings into themes.

**Results:**

Six themes that reflected HCPs’ experiences with the course were constructed: (1) the nature of working in rehabilitation; (2) different perspectives on burnout in specialist rehabilitation; (3) a new perspective, less self-criticism, more self-compassion; (4) growing recognition of the importance of compassion for oneself and others; (5) challenges engaging with the SCHC course; and (6) challenges to sustaining self-compassion in specialist rehabilitation.

**Conclusions:**

Specialist rehabilitation HCPs who participated in the SCHC course developed a new understanding of the importance of fostering self-compassion and compassion for others. This shift may have supported HCPs in navigating workplace challenges, and HCPs described experiencing changes that could help with burnout, improve their care of patients and relationships with colleagues.

## Introduction

The ability to reach person-centered rehabilitation treatment goals relies on effective longitudinal communication and relational collaboration among patients, families, and health care providers (HCPs), and in this context HCP compassion is particularly important [1-3]. Compassion from HCPs enhances patient satisfaction, treatment adherence, and goal achievement [1]. Compassion describes sharing another’s feelings coupled with a desire to relieve distress by taking action [2]. Compassion is often overlooked as a component of care in acute settings, but may be more prominent in rehabilitation settings due to the focus on each individual’s recovery of independent function [2]. Indeed, a review of therapeutic interventions revealed that compassionate care is valued by people undergoing rehabilitation and their healing is promoted by a comforting environment [4].

Ensuring that compassion features routinely in specialist rehabilitation is challenged by HCP compassion fatigue [1], burnout [5], and secondary traumatic stress (STS) [6-11]. HCPs working in rehabilitation often endure an emotional burden throughout prolonged patient stays, especially as they manage patient and family expectations [12,13]. Compassion fatigue, a state where HCPs experience the reduced ability or motivation to care for their patients due to the sustained stressors of their duties [14,15], negatively impacts HCP quality of life and well-being [16]. Burnout is a linked construct characterized by emotional exhaustion, objectification of patients, and a reduction of effectiveness at work [17]. STS refers to a scenario where HCPs may develop symptoms typical of posttraumatic stress disorder, which mirror those of their patients, and arises when HCPs work with people who have endured traumatic events [6]. These constructs are highly correlated [18], but most research has focused on the effects of burnout, with well-developed literature on drivers and consequences [19,20]. Unfortunately, the COVID-19 pandemic and rise of digital health care in Canada have increased HCPs’ risk of experiencing compassion fatigue, burnout, and STS [21,22].

To mitigate negative effects on HCPs, interventions focused on stress reduction and increasing resilience have shown effectiveness for reducing burnout and STS [23]. Mindfulness-based interventions (MBIs), which aim to systematically teach participants to “pay attention, in a particular way, on purpose and nonjudgmentally” have been widely used and shown to improve stress levels and reduce anxiety and depression among HCPs. While MBIs are helpful in mitigating the effects of burnout in-person [24] and online [25], experiences have not been assessed in rehabilitation settings. MBIs may not be feasible for busy HCPs as they are usually conducted over 8 weeks. Evidence suggests that MBIs may be the most beneficial (eg, accounting for reductions in stress) and the best attended in-person or synchronously online [3,26,27] and that brief, informal sessions (eg, less than 4 hours) improve accessibility [28]. Although the optimal MBI delivery method for HCPs is unknown, the combination of compassion cultivation training and mindfulness techniques may help mitigate the effects of burnout, compassion fatigue, and STS [29,30].

Like MBIs, compassion-based interventions effectively reduce anxiety, depression, and psychological distress, but they differ by placing greater emphasis on fostering supportive relationships and common humanity [31]. Similarly, self-compassion interventions also reduce anxiety and depression [32], and were found to do so to a greater extent than MBIs in a 2018 review [33]. In fact, an analysis of a large cross-sectional survey of nurses revealed that self-compassion was protective against burnout-related reductions in compassion [34]. Compassion-based interventions may be better than MBIs at improving HCP well-being and compassion [32], but they are complex interventions and the optimal delivery method is also unknown.

It is crucial to consider the digital landscape in which Western health care is situated with the rise of telemedicine and frequent use of digital technology since COVID-19 [35]. A mix of “asynchronous” (prerecorded online) [36] and “synchronous” (live online) components may best fit the needs of busy HCPs whose personal and professional duties require increasing levels of engagement with online platforms. An intervention which can be accessed “whenever and wherever” is likely to increase accessibility and thus improve engagement [25]. Indeed, the rise of digital health care may facilitate the success of MBIs delivered online rather than in-person [37]. A 2024 narrative review of randomized controlled trials assessing the efficacy of digital mental health interventions for HCPs revealed positive effects for targeting burnout, and acceptability was reported [38]. Factors such as flexibility, ease of use, the availability of support and feedback, and the likelihood of achieving positive outcomes were identified as facilitators of intervention effectiveness [38]. Furthermore, a 2024 scoping review on the assessment of audio-guided online MBIs suggested the need for future research to assess the diverse methods of online delivery (eg, videos and podcasts) [25].

Therefore, the purpose of this study is to explore the experiences of specialist rehabilitation HCPs with an online Self-Compassion for Healthcare Communities (SCHC) course [32].

## Methods

### Study Design

#### Overview

This study presents the qualitative process evaluation of a linked feasibility trial testing the SCHC course in specialist rehabilitation providers. No quantitative trial data are presented in this manuscript. Specialist rehabilitation providers are defined in this context as regulated health professionals working in “specially trained interprofessional teams with a focused skill set,” [39]. The SCHC course on which participants were interviewed was delivered over Zoom (Zoom Video Communications, Inc), with weekly 60-minute sessions on Fridays between 12 PM and 1 PM, by a certified SCHC instructor (a specialist neuro-occupational therapist) and assistant (trainee clinical psychologist), with participants attending whole group sessions and regular smaller group break-out sessions. Participants were assigned home practice activities that varied weekly. An overview of intervention content and week-by-week session guide is available in Multimedia Appendix 1.

Individual postcourse interviews were conducted by the study coordinator on Zoom. The Zoom platform was chosen due to widespread use across the institutional settings involved in recruitment, and to stay consistent with the course delivery. In addition, research participants generally view online interviewing methods for qualitative studies favorably, even over in-person interviews [40,41]. In-depth semistructured interviews were conducted following the final SCHC session. The interview topic guide (see Multimedia Appendix 2) explored participants’ experiences with the SCHC course, its perceived effects, usage, barriers and facilitators to participation, and suggestions for improving future iterations.

#### Theoretical Framework

A qualitative interpretive methodology with a reflexive thematic analysis design and an inductive approach to theme generation was used without relying on preexisting theories or frameworks [42,43] to explore participants’ experiences with the SCHC course. This methodology was selected to learn from qualitative data directly and to highlight participants’ experiences. An inductive, rather than a theoretical analysis, relies on researchers’ interpretations of the raw data, which may stray from the specific questions posed to participants [44].

As a reflexive thematic analysis, the aim was to achieve “information power” with an emphasis on data quality rather than data saturation, which prioritizes data quantity [45,46]. Whereas data saturation occurs when no new themes arise from coding new information gathered from the sample, information power refers to “the richness and relevance of data collected from a sample” [47]. Aiming to achieve a level of meaning in the data (ie, information power), and not solely redundance, is consistent with the method of a reflexive thematic analysis [45,46].

The COREQ (Consolidated Criteria for Reporting Qualitative Research) checklist was followed to ensure ethical data collection and rigorous reporting methods (see Checklist 1) [48].

#### Participant Selection

Eligibility criteria for the feasibility trial in which this study’s data were drawn from were being an HCP, 18 years of age or older, working substantially (ie, not agency staff) in a specialist rehabilitation setting (in-patient and out-patient) across existing specialist rehabilitation sites in a large metropolitan area (ie, the Greater Toronto Area), having the ability to communicate in spoken and written English, and not having undertaken a mindful-self-compassion course or other MBI in the past 12 months. All participants in the SCHC course (n=26) were eligible to interview. Participants were informed they would be invited for an interview following the course, but that participation would be voluntary. Following the course, all enrolled participants received up to 3 successive email invitations to schedule an interview with the study coordinator.

#### Data Collection and Setting

Demographic data (eg, age, gender, and professional role), available in Multimedia Appendix 3, were collected prior to course commencement via an online survey. Interviews were held and recorded on Zoom by the study coordinator (EC), who was present throughout the course only to manage administrative work and technology issues. On average, interviews lasted 50 minutes. Virtual interviews were hosted from the study coordinators’ place of work. Participants joined their one-on-one interview from their workplaces or their homes. The interview audio files were transcribed verbatim by a secure third-party service and not returned to participants for review.

### Research Team and Reflexivity

#### Personal Characteristics and Relationship With Participants

The research team situates themselves within a pragmatic paradigm, centering their beliefs and motives on the real-world significance and usefulness of research findings (ie, what is practical and achievable). In this case, this applies to the SCHC course as a tool for helping specialist rehabilitation providers address the well-documented challenges of busy and stressful frontline clinical care [49].

The lead author (EC) is a younger Canadian woman and an early career researcher. Her undergraduate and postundergraduate experience with qualitative research made her well-suited for involvement in this project while bringing a fresh lens to the analysis as a student who studied psychology in undergrad and studied aging and health in graduate school. Without firsthand experience as an HCP, previous knowledge of the SCHC course, and prior relationships with participants, she was well-placed to immerse herself in the data while maintaining a broader perspective of HCP experiences with burnout. The lead author (EC) conducted the interviews to limit influence on participants as she is not an HCP herself. The lead author (EC) was known to the participants as the study coordinator, but she did not provide any of her own personal insights throughout the course or during the interviews.

The senior author is a middle-aged Scots Canadian man who works as a physiatrist with a research background in developing and evaluating complex interventions, including those based in mindfulness and compassion. His holistic understanding of working in chronically stressful health care environments and his firsthand experience with burnout among fellow HCPs in diverse settings provided crucial insider perspectives, shaping how burnout was conceptualized and interpreted. To minimize potential influence, the senior author did not attend the sessions or conduct the interviews.

The third researcher (KMK) involved with data analysis (female) is a research associate experienced in qualitative research and thematic analysis. Her background in developing and evaluating complex interventions, including those mindfulness-based, provided a valuable lens to data interpretation. Additional authors are researchers and clinicians in rehabilitation science providing guidance and expertise in the design, conduction, and review of this project.

#### Reflexivity

The research team included a diverse range of perspectives and backgrounds, and this richness of experience and diversity was leveraged during the interpretive process with reflexivity practiced throughout. For example, the lead author (EC) kept a reflective set of notes of her thoughts and experiences of sitting-in on the SCHC course, during the semistructured interviews (which she conducted), when working through the development of a theme journal (ie, a flexible and evolving analytic tool used to keep track of theme development), and shared these reflections with the team during regular collaborative meetings where a rich discussion and a deeper exploration of the meaning of data was strongly encouraged. Meeting notes were made and continually updated as data were qualitatively coded and themes were developed. The primary investigator of this study is a physician and how this perspective, and associated power dynamics, might influence views were transparently acknowledged and discussed throughout the entire reflexive thematic analysis process. For example, team members (KMK, DL) with clinical expertise noted their inclination to prioritize certain health-related aspects of what participants described, while others from research-focused roles highlighted broader social and cultural contexts within the story being developed.

### Data Analysis

Data were analyzed using a reflexive thematic analysis design with an inductive approach [44,50]. The steps followed were:

1. Familiarization: a total of 3 researchers (EC, KMK, and RobertSimpson) situated themselves with the deidentified interview data by reading transcripts several times and noting patterns or interesting points. The involvement of multiple researchers enhanced analytic depth and fostered reflexive dialogue, which was apparent throughout the entire thematic analysis process.
2. Initial code generation: initial codes were synthesized from each interview transcript in NVivo (version 12; QSR International Pty Ltd). Through an iterative coding process, 2 researchers labeled meaningful chunks of data with phrases that aligned with the research objective (eg, “course format was accessible”). A team meeting was held to facilitate collaborative discussion of initial codes, groupings, and patterns within the data. This discussion led to the re-organization of some groupings to maintain relevance to the research objective while assumptions were questioned. Initial code generation continued until all transcripts were fully labeled and organized, which itself was an iterative and recursive process.
3. Initial theme generation: subsequent team meetings facilitated theme development through discussion, during which initial themes (eg, “patterns of shared meaning” [51]) were constructed by grouping initial code labels together based on similarity and naming these groupings in NVivo. Preliminary theme definitions were also synthesized. Initial theme names and definitions were noted in a shared theme journal document to keep track of meeting progress. At this stage, mind maps were also created and continuously revised during and between meetings on an online design platform to assist with visualizing the meaning of initial groupings and the connections between them, while being able to zoom out and grasp a holistic view of the data.
4. Reviewing and developing themes: several weekly meetings were held to continue developing themes through collaborative discussion of the patterns within the data, ensuring that meaningful insights were representative of the groupings. Continuous reflexive engagement supported the development of cohesive storylines for each theme.
5. Refining, redefining, and naming themes: the research team iteratively refined the theme journal during regular conversational meetings by revisiting themes to ensure relevance to the objective. This process involved ongoing iterative refinement of themes and definitions by continuing reflexive engagement with the data. Researchers continued to question their initial assumptions and interpretations of the data and always attempted to view the data from several stances. Theme names were finalized based on theme definitions. Updated mind maps helped researchers visualize the story told by the data.
6. Producing the report: the analysis was written up to present a coherent story of the data, with continued reflexive engagement guiding development. Direct quotes from participant interviews were extracted, supplemented with researchers’ careful interpretations of each theme and how they connect to one another. Findings were situated among current knowledge in the field.

### Ethical Considerations

Ethical approval was obtained from Sunnybrook Health Science Centre Research Ethics Board (SUN-5492). All study participants provided prospective written informed consent.

## Results

### Overview

**Figure 1.**
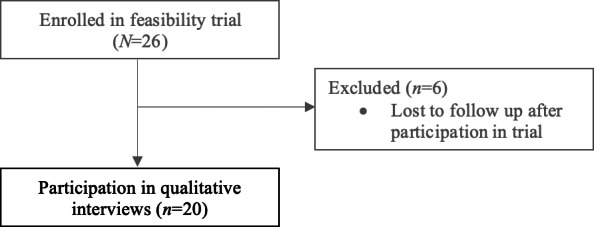
Participant flow diagram. Gray represents the linked feasibility trial in which this qualitative study draws data from. Detailed trial feasibility metrics are reported separately (under review).

Rehabilitation providers (n=20; see Multimedia Appendix 3 for participant demographics) who engaged in the SCHC course shared their experiences working in rehabilitation as well as their experiences with the course, thus resulting in 6 interconnected themes that portray HCPs’ intricate experiences. Only a subset of participants was interviewed, and we do not have information on the reasons why some individuals chose not to participate despite efforts to follow up after participation in the course (see Figure 1). This may have introduced self-selection bias, as those with more positive experiences may have been more likely to take part in interviews. The mean (SD) age of participants was 43.5 (10.5), the majority identified as women, almost one-third identified as White North American, and 96% worked in urban environments whereas one participant worked in both urban and rural areas. For an average of 13.6 years, HCPs worked primarily as occupational therapists and social workers, but there was indeed a vast spread of professions within specialist rehabilitation represented.

Participants spoke of (1) the nature of working in specialist rehabilitation, an interplay between longitudinal relationships and focus on adaptation, which heavily impacted (2) different perspectives on burnout in specialist rehabilitation and experiences with the course. Throughout the course, (3) a new perspective, less self-criticism, more self-compassion, was gained by many participants as they learned how to alter their self-deprecating attitudes. Once self-compassion was fostered using various techniques learned throughout course sessions, (4) growing recognition of the importance of compassion for oneself and others arose as participants learned how to extend their newfound sense of self-compassion to their patients, family members, and colleagues. Unfortunately, (5) challenges engaging with the SCHC course were inevitable as HCPs endured busy schedules and individuals varied in their comfortability being vulnerable in an online, group setting alongside their peers. Although it was reported by participants that the course helped cultivate self-compassion and enhanced their compassion for others, several factors limited the ability of HCPs to extend this compassion to their patients. These factors are described in the last theme: (6) challenges to sustaining self-compassion in specialist rehabilitation. Supplemental quotes categorized by theme are provided in Multimedia Appendix 4.

### Theme 1: The Nature of Working in Rehabilitation

The first theme, the nature of working in rehabilitation*,* provides context into the aspects of working in rehabilitation that can render HCPs at risk of compassion fatigue, burnout, and STS and how these factors mirror and differ from those faced by HCPs that work in other settings. Reflecting on their experiences prior to taking the course, HCPs described how they are challenged with distinct stressors due to the nature of the specialty. Rather than curing, HCPs in this field are tasked with helping their patients adapt to new circumstances, often following life-changing illness or injury. Often, patients in rehabilitation are perceived by HCPs as initially resistant to adaptation as their expectations had not been addressed in acute care. Due to the nature of rehabilitation, HCPs build long-term relationships that can last from weeks to decades. HCPs may also connect with their patients on an emotional level, which can have positive and negative implications for well-being. Some HCPs endure long-term maltreatment from patients, and others are affected by the implications of difficult cases outside of working hours.

*I only recognized in the last couple years … a lot of abuse and dumping … negative energy put onto you. People … or my other colleagues don’t realize after four to six weeks of someone being so negative, there is an impact on you*.

[Woman, aged 45 years]

*I would be left thinking about the traumas that they had experienced…And worrying if they’d be alive tomorrow…It was harder to disconnect…I wouldn’t be working, but the thoughts would still be there, or I’d have nightmares*.

[Woman, aged 30 years]

Like other HCPs, rehabilitation providers experience occupational stress from a lack of agency, large caseloads, staff shortages, time constraints, and excessive administrative work. Rehabilitation HCPs described how they often experienced moral distress when discharging patients to what were perceived to be unfit home environments, simply to meet organizational pressures around bed occupancy. Stress was accentuated for specialist rehabilitation HCPs when required to perform their duties, and sometimes colleagues’ duties, within an overwhelmed system.

*Some days are stressful because I’m asked to see four patients at a time. And there’s no helping around. Or…there’s three patients in the gym that I have to leave unattended… the nonsense that goes on around here is frustrating beyond*.

[Woman, aged 34 years]

These stressors, some of which may be unique to rehabilitation, some which are common throughout health care, made the SCHC course relevant for participants as they sought out strategies to manage burnout and build resilience.

### Theme 2: Different Perspectives on Burnout in Specialist Rehabilitation

Participants from different backgrounds and professional roles described the concept of burnout in various ways, whether they had learned about the concept from the course or elsewhere. Participants described experiences with burnout affecting numerous aspects of their lives, especially in relation to their work in rehabilitation.

Participant feedback suggested the SCHC course might help improve participants’ abilities to recognize and describe burnout and compassion fatigue, phenomena that were described as common. Indeed, burnout was described by participants as poorly functioning or performing at work. Accordingly, burnout was described as affecting more than one dimension of a person’s life, including emotionally and physically, which was seen to impact participants’ personal lives in an “…exponential downward spiral…” (Man, age 35 years).

*Burnout is being both emotionally, physically…spiritually, like just out of steam, and not able to get through the day without interventions. So, like there was a time when I was burned out and I needed to take naps during the day. I just was emotionally exhausted and in the afternoon I would take two hours out of work, and then I would work later, because I needed that energy to keep going*.

[Woman, aged 50 years]

Participants discussed burnout and compassion fatigue as distinct but related concepts. Specifically, participants described compassion fatigue as a concept constituting a precursor to burnout, which encompasses emotional exhaustion and limits the quality of compassionate care provided to patients.

*It starts to weigh you down, especially when you hear some of the stories. Because the patients, they open up to you … they share so much of their life experience. And you listen with compassion. But it does wear you down after a while*.

[Woman, aged 34 years]

Through attending the course, participants explained gaining a clearer vocabulary to describe burnout and compassion fatigue, enhancing their self-awareness and recognition of these phenomena in their daily practice.

### Theme 3: A New Perspective, Less Self-Criticism, More Self-Compassion

The third theme speaks to gaining a new perspective when it came to teaching participants how to cultivate more self-compassion and less self-criticism. Participants recognized the importance of incorporating this new perspective to shape their attitudes about themselves and others in a positive way.

Following the completion of the course, most participants shared that the concept of self-compassion had been new to them. Most described being conscious of compassion for their patients, including the circumstances which would activate this way of being, but acknowledged not recognizing ever being compassionate to themselves. A common description across interviews was that self-compassion is more difficult to inculcate than compassion for others,

*It’s easy just to blame yourself. Self-compassion I find is harder. I find it much easier to be kinder to others than to myself.*

[Woman, aged 49 years]

Frequently, providers described putting patient needs and work tasks before their own. Participants often recognized a tendency to self-criticize and be hard on themselves.

*But I do think I’ve had difficulties with self-compassion because I grew up in a very domineering system. You’re either wrong or you’re wrong. You’re never right. And with that, I’ve internalized a lot of that*.

[Man, aged 35 years]

Despite this, participants described curiosity about the concept of self-compassion, seeming to recognize a clear need for self-care and lacking a resource to do so.

*I didn’t know what to expect, because I think when I hear the word self-compassion, my mind can immediately go to, oh, get a massage, pamper myself…So, I was definitely curious as to what would come out of this. I was pleasantly surprised. I just went in with an open mind. But I think I took away something more than I thought I was going to take away*.

[Woman, aged 44 years]

Participants reflected on gradually learning to recognize self-critical tendencies throughout the course and beginning to adopt new approaches over time, with deliberate attempts to incorporate compassionate responding to themselves. Recognizing the adverse effects of self-criticism, participants began to see self-compassion as humanizing how they responded to themselves.

*Self-compassion means having compassion for yourself as an individual…This is something I really resonated with in the course and thought about it in a new way. It’s like just feeling. Just remembering that you are a person too…Recognizing it’s something I have to continue to work on from the course*.

[Woman, aged 60 years]

Participants also noted that this had beneficial feed-forward effects in relation to others through common-humanity, with a renewed desire to alleviate the distress that all humans experience.

*My peace can be restored when I remind myself that I’m not the only person navigating these feelings and thoughts. That common humanity piece is really important to me because it makes me feel less isolated*.

[Woman, aged 49 years]

### Theme 4: Growing Recognition of the Importance of Compassion for Oneself and Others

Participants described that recognizing the importance of compassion for oneself and others became more apparent throughout the course as they reflected on learning how to let go of their self-critical attitudes over time and focused on being kind to themselves, using the learned self-compassion practices, and listening to peers’ similar experiences.

Participants described that a fundamental premise to fostering self-compassion is recognizing it is okay be kind to oneself, even as an HCP “it’s okay sometimes to just step back and take care of yourself first, before other people,” (Woman, aged 53 years). Participants reflected on using self-compassion practices more naturalistically in their day-to-day lives as the course progressed while others described using what they had learned to incorporate in their clinical care of patients, or in their professional lives with students, or in their personal lives with family members.

*I use the [hand on heart strategy]. I’ve been doing that with my family too. Like my wife when we talk, just to remind myself to be grounded. And just the practice of doing the exercises I think just made me more attuned to my patients and just helped me remember to hold space for people and have more patience. Immediately, I just felt like my practice was shifting a little bit*.

[Man, aged 43 years]

Participants iterated a perceived importance of regularity of the self-compassion practice as a means of developing the skill and driving lasting change. In this regard, the course environment appeared to motivate continued engagement with the self-compassion practices, where participants described being inspired by others’ reflections on personal insights and use.

*I really did appreciate listening to others. And I found some of the other participants were so thoughtful and open and vulnerable and really shared both their struggles and how the practices were really meaningful to them. And that was inspiring in a way, to know other people are also struggling similarly*.

[Woman, aged 42 years]

Indeed, the online nature allowing providers from otherwise separate rehabilitation sites reinforced a sense of camaraderie and solidarity.

*A year or so ago, a patient died… We don’t get a lot of deaths in rehab. And in rehab we’re not trained for that. Being able to sit down as a group after that happened, no management, just us, and talk about how we were feeling from everybody’s perspectives… was the most important thing for us to prevent burnout… and all help each other get past it*.

[Woman, aged 40 years]

Setting work-life boundaries, “not taking any work home” or “not looking at emails*,*” (Woman, aged 26 years) was also deemed a crucial element to self-compassion by participants, who described this as necessary to maintain their own well-being and ability to provide compassionate care to patients. According to HCPs, developing an understanding of cultivating inward compassion, through learning and applying specific strategies, played an integral role in fostering a grounded and clear mindset needed to extend compassion toward others.

### Theme 5: Challenges Engaging With the SCHC Course

Participants described several barriers to course participation from the method of course delivery to challenges with course content, as well as individual variation in level of readiness to engage in sensitive conversations. A striking insight shared was that to make time for course engagement, participants had to give up time spent charting during their lunch-hour (ie, investing time in themselves came at a cost, causing anxiety surrounding completing work later in the day.)

*I didn’t attend all the courses. I think I only attended two and then I really had to back out because it was causing a lot more stress in terms of my workload, and I sat there more anxious than anything, so it was setting my afternoons off*.

[Woman, aged 45 years]

Other challenges noted included a sense of discomfort in connecting emotionally to others online and participants worried about how exposing oneself in this way might undermine their professional identity,

*When you’re not surrounded by people, physically, you can’t pick up on their body cues. It’s just not a very comfortable environment to talk about your emotions or talk about your feelings*.

[Woman, aged 34 years]

While some HCPs found aspects of the course to be conducive to engagement (eg, the diversity of attendees and the online format), there were many suggestions for improvement which primarily focused on timing, mode, and content. Suggestions did not follow a specific pattern and varied from implementing an asynchronous course to hosting the meetings before working hours to limiting the group to a smaller size. A persistent suggestion was tailoring content to typical challenging rehabilitation scenarios and implementing a follow-up course to reinforce new skills.

### Theme 6: Challenges to Sustaining Self-Compassion in Specialist Rehabilitation

Several barriers to being compassionate in rehabilitation beyond course completion were perceived, ranging from individual (eg, changing habits) to systemic (eg, heavy workload) in nature. However, following course completion, participants explained that by necessity they learned how to implement self-compassion practices from the SCHC spontaneously in their daily lives despite obstacles, such as stepping aside for a brief period to “ground” oneself during unbidden stress.

Perceived role stipulations were cited to challenge practices from the SCHC in specialist rehabilitation, where an explicit emphasis is placed on HCPs prioritizing physical function, and less attention paid to emotional distress which often underlies impairment and impedes functioning.

*I would say most people who are struggling physically are also struggling emotionally. I found often with [stroke patients], it was harder to get a read on really how much someone was struggling, especially if their speech was impacted. It was a little bit harder to connect with people because you couldn't quite tell all the time how much emotionally this has impacted*.

[Woman, aged 40 years]

Participants also noted that implementing practices from the SCHC course could not address prevalent systemic issues directly, such as when patient and family expectations for rehabilitation were perceived as unrealistic due to not being addressed in the acute setting,

*So that, I find, has always probably there been a consistent stress factor, dealing with families with high expectations and then managing their expectations. So, when you have those conversations and if they're very anxious or stressed and they come at you and they use you as a scapegoat sometimes*.

[Woman, aged 44 years]

Systemic-level realities such as large caseloads, time constraints, and the fast pace of inpatient units were all seen as impediments to maintaining compassion, despite learnings from the course. Hinting at moral distress, there were clear depictions of the struggle to balance personal capacity (eg, energy) for delivering exceptional, compassionate care with the relentless demands of the work environment. This finding underscores the necessity of learning and implementing tools to foster self-compassion in an environment that may not inherently promote it.

*I’m struggling with taking care of a lot of patients. I often-times feel like I don’t give enough attention to them. It’s more about I’m feeling guilty if I don’t give enough attention to them, and I know, if I have the time, I can make a lot of difference. But I just cannot. And after all these years I do make sure I don’t spend way too much time here. I know I can’t sustain that*.

[Woman, aged 59 years]

## Discussion

### Principal Findings

In this exploratory qualitative study, specialist rehabilitation providers were provided with the opportunity to engage in the SCHC course online. Reflexive thematic analysis generated six prevalent and interconnected themes: (1) the nature of working in rehabilitation, (2) different perspectives on burnout in specialist rehabilitation, (3) a new perspective, less self-criticism, more self-compassion, (4) growing recognition of the importance of compassion for oneself and others, (5) challenges engaging with the SCHC course, and (6) challenges to sustaining compassion in specialist rehabilitation.

The nature of working in rehabilitation highlights the complexities of specialist rehabilitation that can render HCPs at risk of compassion fatigue, burnout, and STS. Not only does the focus of specialist rehabilitation—adaptation—contrast with the focus of acute care*—*treatment or cure*,* but also patient and families’ expectations of the goals of rehabilitation are rarely managed beforehand. Thus, managing expectations following a disabling life event becomes a daunting and burdensome task for HCPs to fulfill that extends beyond dealing with the emotional distress that may arise from completing their regular tasks. Additionally, the longitudinal nature of specialist rehabilitation affects HCPs in 2 capacities. On one hand, HCPs describe the value in getting to know their patients on a deeper level and the satisfaction of helping patients improve their functioning throughout time. On the other hand, HCPs may endure long-term maltreatment from their patients and their families if expectations are not managed properly and if functional improvements are not apparent. Further, these effects may persist outside of working hours, affecting HCPs in profound ways. Although familiar for specialist rehabilitation HCPs, these nuances may not be well understood among the general public [52,53], and effects on HCPs’ well-being are not always incorporated into organizational guidelines or well-documented in the literature [54,55].

The nuances that a specialist rehabilitation setting offers may expose HCPs to highly traumatizing experiences [8] which may contribute to a significant risk of burnout [56]. Most participants in this study were unclear about how to define or differentiate between compassion fatigue, burnout, and STS. Many had heard of the concept of burnout before the course and could speculate at its meaning based on personal or colleagues’ experiences. Most conveyed experiencing stress through providing patient care, some sounding personally traumatized. Participants discussed the course helping to grow their understanding of the concept of burnout and that it also provided a safe forum for discussing difficult experiences among peers who could understand, to the extent that this fed into suggestions for making the course even more relevant. Elsewhere, other self-compassion intervention studies have quantitatively reported reductions in symptoms of burnout among HCPs following participation or in comparison to a control group [57-60], and have also reported that HCPs developed an increased awareness of their own well-being [58]. In this exploratory qualitative reflexive thematic analysis, we did not assess quantitative changes in burnout pre- to postcourse, but participants did report that through participation in the SCHC course they became aware they may be experiencing burnout and also gained useful tools to combat these challenges. A better understanding among HCPs of how work in specialist rehabilitation can affect one’s occupational well-being may facilitate engagement with evidence-based self-care strategies and may feed into concerted efforts to change a culture in health care that raises the risk of burnout for many HCPs [61].

HCPs have ethical duties to do no harm and to act in patients’ best interests (eg, [62,63]), even if this means doing so at the expense of their own well-being, which is a prevailing and problematic cultural norm in health care [32]. Therefore, it is not surprising that participants described having an easier time being compassionate to their patients than to themselves. Most HCPs recognized that they are regularly compassionate to their patients yet had never labeled their actions as such. Even fewer participants acknowledged intentionally being self-compassionate while reflecting on their lives prior to the course. While the course attempted to emphasize the opposite, for many participants, to identify professionally as a specialist rehabilitation HCP, one cannot also show humanistic qualities. Whether this belief was perpetuated by personal factors or systemic norms and the complex association between necessitates future research. Being a “human being” (eg, being exhausted or not performing your best at work) lends well to practicing self-compassion, but for HCPs, having self-compassion was not possible without compromising professional identity. Accordingly, it appeared to take time for HCPs to gain a new perspective, less self-criticism, and more self-compassion to challenge their beliefs. In line with the findings of Neff et al [32], the SCHC course may be more beneficial for HCPs who report having low self-compassion to begin with, although in this study, changes over time were described retrospectively and not formally assessed longitudinally.

Following the new perspective participants described, many discussed the course being helpful in growing recognition for the importance of compassion for oneself and others. Participants found being intentional about one’s thoughts, feelings, and actions important when seeking to cultivate compassion and debriefing about difficult situations as a health care team an important mechanism to bring compassion to bear on collective adverse experiences. Descriptions of growing compassion may align with current quantitative evidence that mindful self-compassion interventions are successful in minimizing HCPs’ self-criticism [59] and improving self-compassion [32,57-60]. This study is the first study to report participant experiences of growing self- and other-focused compassion in specialist rehabilitation HCPs, despite these constructs not being assessed quantitatively.

The “new” concepts covered in the SCHC course were well-received by HCPs who were open to learning about how to improve their well-being by increasing their self-awareness and engaging with self-compassion practices. Similarly, existing literature affirms the importance of practicing the techniques from the SCHC course in a health care setting for oneself and for one’s patients [58,59]. Accordingly, promoting common humanity [64] and fostering communities of support [58] continue to be useful tools to promote compassion in HCPs.

HCPs provided both positive feedback and constructive criticism based on the delivery of the SCHC course. The online format provided participants with the flexibility to join from several sites across a large metropolitan area, and the diversity of professional roles was appreciated and contributed to a sense of common humanity when peers discussed similar experiences in their duties even across sites. Additional advantages that supplement previous research [57] include saving travel time and facilitating joining from home. One of the most notable suggestions was to tailor the content to include rehab-specific examples. This suggestion was also reported in recent reviews. Emphasis on the importance of contextual adaptation underscores the need for tailoring digital interventions to the specific group of HCPs they intend to be delivered to [38] (eg, rehabilitation providers). A 2024 scoping review specific to the assessment of online MBIs primarily using audio-guided programs also suggested tailoring interventions for groups of HCPs [25]. This change is likely to enhance acceptability but would necessitate further feasibility testing.

Despite participants’ acknowledgment of the benefit of self-compassion practices they learned, many noted challenges engaging with the course such as scheduling challenges, expected with busy HCPs [32]. Interestingly, not being ready to share personal struggles in a group setting was a less common challenge. It is possible that those who were unwilling to share did not participate in interviews entirely. The fact that some HCPs disclosed these feelings still emphasizes participants’ curiosity about the self-compassion intervention and the importance of having trained facilitators to handle challenges that may arise. While the group format (eg, the presence of unfamiliar peers) hindered some participants’ willingness to share and reduced their ability to build rapport with other course participants and facilitators, the added benefit of accessibility must be weighed with the impracticalities of in-person sessions. However, most participants appeared to value the format of the course. Also noted was the impracticability of engaging in self-compassion practices and actions at work, in line with Crandall et al [58] and in contrast to Delaney’s [59] findings that most nurses found their self-compassion practices useful on the job. The lack of suitable work environments to practice self-compassion (eg, a quiet location to engage in the “hand on heart” practice) speaks to the need for leaders and organizations to understand the need to focus on employee well-being and appropriate infrastructure change (eg, staff “recovery” rooms; [4]).

To provide compassionate care to promote better patient satisfaction [1] and healing [4], both individual and systemic factors should be targeted. Participants spoke of their complex patient populations and difficulties managing patient expectations to limit the compassion they were able or willing to give. Patients undergoing specialist rehabilitation often exhibit distress in more than one area of their lives (eg, physical and emotional) as they typically experience life-altering diseases or catastrophic injuries [65,66]). HCPs should be aware that patients are frustrated with their new realities or the health care system more generally, which can be perceived as dehumanizing both by HCPs and patients alike [67]. Speculatively, increased awareness of shared distress and common humanity through participation in the SCHC course may limit HCPs’ internalization of patient frustration, to instead respond with compassion, and this in turn may limit their risk of feeling burnt out, although this remains speculative and would have to be tested quantitatively to map out processes and determine proponent causality. Regardless, HCP training in compassionate care should arguably include strategies for helping identify the source of patient frustration and provide tools for HCP emotional awareness and regulation. Although not only rehab-specific [68], systemic challenges that feature in HCPs’ day-to-day include being overwhelmed with case numbers and not having enough time to complete their duties. From participant accounts, the guilt that comes with wanting to be compassionate, but not being able to, may perpetuate specialist rehabilitation HCP moral distress and feelings related to burnout, and the cycle is likely to continue if changes are not made. Policy changes facilitated by leadership that foster a sense of shared humanity and a healthy workplace environment are likely to tackle barriers to compassion, such as implementing protected time with management to reflect on challenges as well as taking short breaks throughout the day [69,70].

### Limitations

Although rich data were gathered to portray HCPs’ diverse experiences with workplace-related stress and the SCHC course, this study comes with limitations. First, generalizability to the population of specialist rehabilitation providers is restricted. The sample all worked in the Greater Toronto Area, which limits applicability of findings to other populations across Canada or countries outside of Canada, especially those from rural areas. Fewer HCPs are employed in rural compared to urban environments, which can intensify workplace stress and increase the prevalence of role sharing among rehabilitation disciplines [71]. Such factors may intensify HCPs’ experiences with burnout and thus, the course may need tailored in relation to the needs of the population served. For instance, rural HCPs may find themselves experiencing more debilitating workplace stress that may or may not classify as burnout, as they may take on additional roles outside their scope of practice or work more hours than urban HCPs. Despite this limitation, the sample was heterogeneous, containing people of various ages and professional disciplines in rehabilitation who attended most sessions. This study recruited participants from a specific geographical area due to the nature of the research protocol. Future iterations should consider expanding the sample to increase generalizability and assess feasibility on a wider scale, something which offering an online SCHC, as opposed to in-person, can help facilitate.

Further, it is entirely possible that participants who did not interview had different experiences with the course than those who did interview, reflecting potential self-selection bias. Common barriers for participation in qualitative research include the time requirement (eg, more time intensive than survey-based research) and lack of incentives [72]. Noninterviewees could have been too busy at work to participate in an interview and thus, they may have described more severe workplace-related stresses than the group who interviewed. All participants were offered gift cards to interview. In addition to time and incentives factoring into one’s decision, those who did not interview may have had poorer experiences with the course than counterparts who interviewed. Alternatively, nonrespondents may have felt as though the course was not relevant to them, which may explain the lack of desire to provide feedback. These characteristics may or may not have altered the themes that arose from this analysis if the nonrespondents provided interview data. Ultimately, it was difficult to conclude why the 6 participants did not choose to interview as they did not respond to our interview invites even after following up. Positive findings, including the perceived growth of compassion and course accessibility, should be interpreted while considering the potential reasons why a subset of participants chose not to interview. Nevertheless, the number of participants who interviewed was high and motivation for participation was captured, important pieces of the puzzle when analyzing acceptability of a program in a specific setting.

Social desirability bias, the tendency to respond in accordance with perceived socially acceptable characteristics or favorable outcomes [73], may have influenced interviewee responses. The more engagement with researchers students in a learning setting experience and the more stress in one’s life (eg, being overworked or experiencing burnout), the more likely participants are to respond to questions favorably, without responses reflecting their true experiences [74,75]. Social desirability bias may explain the tendency for participants to report that the SCHC course was helpful in growing their understanding of the concept of self-compassion or limiting feelings associated with burnout or workplace stress. Additionally, HCPs may fear disclosing burnout-related feelings due to stigma surrounding mental health issues, worries about it impacting their employment [76], or privacy concerns [77]. While semistructured interviews were used to gather responses, which may increase the effects of social desirability bias, the study coordinator limited her involvement during the trial to limit potential influence, being on camera only when necessary to solve technical issues or answer logistical questions. Furthermore, the purpose of the study was communicated to participants as not to solely provide tools to improve emotional well-being, but rather to gain insights into the experiences of engagement with the course. Questions were phrased from both sides (eg, in which ways was the course helpful or unhelpful?) or as neutral as possible, and participants were assured their responses would remain anonymous. Social desirability bias has been shown to be minimized in these ways (eg, [73]) and the mix of positive and negative responses, including suggestions for course improvement, help demonstrate this. Aside from the risk of socially desirable responding, online interviewing is liked by participants, easy to use by researchers and facilitators, and conveys perceptions of empathy and rapport well when used for qualitative research [40,41,78].

### Implications and Future Directions

Participation in the online SCHC course appeared to help improve HCPs’ knowledge of the concept of burnout, validate common struggles with their peers, and teach them tools to foster self-compassion and compassion for others. Some participants experienced challenges feeling connected to the course and fellow participants because of the online synchronous format, course timing, and other barriers described. Accordingly, participants described program strengths and areas for improvement. Both positive feedback and constructive criticism provided by HCPs can inform the development of knowledge translation tools (eg, a website), educational sessions, organizational strategies to better support staff well-being.

This is the first study exploring HCPs’ experiences with the SCHC course in a specialist rehabilitation context. Variations of the course should be tested iteratively for acceptability, accessibility, and efficacy by adjusting course timing (eg, time of day), format (eg, synchronous), and class size in line with suggestions provided by our participants. The most prominent suggestion was to make content rehabilitation specific. Future trials should include concrete examples of stressors that may arise in rehabilitation when discussing the need for self-compassion so that participants can relate. Course delivery conditions also matter; protected learning time and continuing education credits may be required.

Accordingly, to better understand potential mechanisms of action and effectiveness (eg, by measuring pre- and postburnout, compassion fatigue, and STS scores), the next phase of research will compare the SCHC course and a matched comparator, such as a Health Enhancement Program. The Health Enhancement Program is an intervention specifically designed to match MBIs in terms of group context, instructor time and attention, education on health behaviors, but not on any content on mindfulness or self-compassion [79]. This would constitute a phase 2 randomized controlled trial design [80]. Additional future studies, such as one that gathers perspectives from health leaders (eg, Division heads) can provide valuable insights into organizational structures that may facilitate participation (eg, the availability of quiet spaces) and clarify responsibility for participation.

### Conclusion

An online SCHC course appears relatively acceptable and accessible to some specialist rehabilitation providers, who noted gaining a deeper understanding of the concepts of self-compassion and burnout and discussed a desire to extend more compassion to themselves, their patients, and colleagues. Barriers to participation, such as difficulties making the time to join sessions and challenges with being vulnerable online were also discussed by participants, which may limit relevance for some busy HCPs. Self-compassion interventions like this one may be a useful strategy for improving HCPs’ well-being in a postpandemic context; however, optimization changes appear necessary to maximize relevance and use for all specialist rehabilitation HCPs, and this should be the focus of future research.

## Supplementary material

10.2196/81706 Multimedia Appendix 1 Week-by-week session guide.

10.2196/81706 Multimedia Appendix 2 Semistructured interview guide.

10.2196/81706 Multimedia Appendix 3 Sociodemographic characteristics of participants.

10.2196/81706 Multimedia Appendix 4 Supplemental quotes.

10.2196/81706 Checklist 1 COREQ checklist.

## Abbreviations

COREQ: Consolidated Criteria for Reporting Qualitative Research
HCP: health care provider
MBI: mindfulness-based intervention
SCHC: Self-compassion for healthcare communities
STS: secondary traumatic stress
