# Supplementary material for: Specialist Rehabilitation Providers’ Experiences With an Online Self-Compassion Course: Reflexive Thematic Analysis
Source: JMIR Rehabil Assist Technol. 2026 Jul 15;13:e81706. doi: 10.2196/81706 (PMC13372217; doi:10.2196/81706)
Supplement: Multimedia Appendix 1 [file rehab-v13-e81706-s001.docx]

**Appendix A**

*Week-by-Week Session Guide*

| Session and Topic | Agenda |
| --- | --- |
| Session 1: What is Self-Compassion? | Topic: Introduction (10 min) |
|  | Topic: How to Approach the Course (5 min) |
|  | Exercise: How Do I Treat a Friend? (15 min) |
|  | Topic: What is Self-Compassion? (10 min) |
|  | Exercise: Hand Gestures (5 min) |
|  | Topic: Misgivings and Research about Self-Compassion (15 min) |
|  | Informal Practice: Self-Compassion Bracelets (5 min – optional) |
|  |  |
| Session 2: Practicing Self-Compassion | Discussion: How is Your Practice Going? (15 min) |
|  | Topic: The Physiology of Self-Compassion (5 min) |
|  | Informal Practice: Supportive Touch (5 min) |
|  | Informal Practice: Self-Compassion Break (15 min) |
|  | Topic: Backdraft (10 min) |
|  | Topic: Mindfulness (2 min) |
|  | Informal Practice: Moments of Mindfulness (8 min) |
|  |  |
| Session 3: Discovering your Compassionate Voice | Discussion: How is your Practice Going? (15 min) |
|  | Topic: Self-Criticism and Safety (5 min) |
|  | Exercise: Motivating Ourselves with Compassion (40 min) |
|  |  |
| Session 4: Self-Compassion and Resilience | Discussion: How is your Practice Going? (15 min) |
|  | Topic: Strategies for Working with Difficult Emotions (10 min) |
|  | Informal Practice: Soften-Soothe-Allow (25 min) |
|  |  |
| Session 5: Self-Compassion and Burnout | Discussion: How is your Practice Going? (15 min) |
|  | Topic: Caregiving Fatigue (20 min) |
|  | Informal Practice: Compassion with Equanimity (25 min) |
|  |  |
| Session 6: Making it Count | Discussion: How is your Practice Going? (10 min) |
|  | Topic: Core Values (20 min) |
|  | Exercise: Setting an Intention (5 min) |
|  | Exercise: What Would I Like to Remember? (15 min) |
|  | Informal Practice: Self-Compassion Stone (5 min) |
|  | Exercise: Closing with Three-Word Shares (5 min) |
