# Supplementary material for: Specialist Rehabilitation Providers’ Experiences With an Online Self-Compassion Course: Reflexive Thematic Analysis
Source: JMIR Rehabil Assist Technol. 2026 Jul 15;13:e81706. doi: 10.2196/81706 (PMC13372217; doi:10.2196/81706)
Supplement: Multimedia Appendix 2 [file rehab-v13-e81706-s002.docx]

**Appendix B**

*Semi-Structured Interview Guide*

1. Can you please describe what compassion means to you?
   1. Can you describe whether/how you were taught about compassion?
2. Can you please describe how you use compassion in your work, and/or give an example?
   1. Can you please describe what you typically think or feel when you perceive that a person is suffering emotionally?
   2. Can you please describe what you typically do when you perceive a person is suffering emotionally?
   3. Can you please describe what you typically think or feel when you perceive a person is suffering physically?
   4. Can you please describe what you typically do when you perceive a person is suffering physically?
3. Can you please describe what compassion fatigue means to you?
   1. Can you describe whether/how you were taught about compassion fatigue?
   2. Please describe any experience you have with it personally?
   3. Please describe any strategies you use to prevent it?
4. Can you please describe a time where you felt compassion was lacking in care you or someone else provided in specialist rehabilitation?
   1. Why do you think this happened?
   2. What do you think might have helped prevent this from happening?
5. Can you please describe what burnout means to you?
   1. Can you describe whether/how you were taught about burnout?
   2. Please describe any experience you have with it personally?
   3. Please describe any strategies you use to prevent it?
6. Can you please describe your views on the emotional burden associated with working in specialist rehabilitation?
   1. Can you please describe how you deal with the emotional burden you experience working in specialist rehabilitation?
7. Can you please describe what you enjoy most about your work in specialist rehabilitation?
8. Can you please describe what you enjoy least about your work in specialist rehabilitation?
9. Can you please describe your best professional achievements?
10. Can you please describe your experiences with the Mindful Self-Compassion (MSC) course?
    1. What worked well?
    2. What didn’t?
11. What are your views on the MSC course being delivered in-person versus online?
    1. Do you think it matters? Why?
    2. Which is preferable to you and why?
    3. Can you describe whether either format would make you more or less likely to use the MSC course?
12. Are there any ways in which the MSC course impacted on your work as a specialist rehabilitation healthcare provider (HCP), positive or negative?
13. Are there any ways in which the MSC course impacted on your relationship with yourself, positive or negative?
14. Are there any ways in which the MSC course impacted on your personal life with others, positive or negative?
15. How do you think the MSC course could be made more relevant for specialist rehabilitation HCPs?
16. Please describe whether you would recommend the MSC course to colleagues, including your reasons for doing so or not?
17. Is there anything else you would like to add?
