# Supplementary material for: Specialist Rehabilitation Providers’ Experiences With an Online Self-Compassion Course: Reflexive Thematic Analysis
Source: JMIR Rehabil Assist Technol. 2026 Jul 15;13:e81706. doi: 10.2196/81706 (PMC13372217; doi:10.2196/81706)
Supplement: Multimedia Appendix 3 [file rehab-v13-e81706-s003.docx]

**Appendix D**

*Sociodemographic Characteristics of Participants*

| Characteristic | *n (M)* | *% (SD)* |  |
| --- | --- | --- | --- |
| Interview participation | 20 | 76.9 | |
| Gender |  |  | |
| Woman | 22 | 84.6 | |
| Man | 4 | 15.4 | |
| Age | (43.5) | (10.5) | |
| Race or ethnicity |  |  | |
| White – North American | 8 | 30.8 | |
| South Asian | 6 | 23.1 | |
| East Asian | 4 | 15.4 | |
| Southeast Asian | 3 | 11.5 | |
| White – European | 3 | 11.5 | |
| Black – Caribbean | 1 | 3.9 | |
| Central Asian | 1 | 3.9 | |
| Rurality |  |  | |
| Urban | 25 | 96.2 | |
| Urban and rural | 1 | 3.9 | |
| Years of experience | (13.6) | (9.0) | |
| Occupation |  |  | |
| Occupational therapist (OT) | 6 | 23.1 | |
| Social worker | 6 | 23.1 | |
| Physiotherapist (PT) | 4 | 15.4 | |
| Physician | 2 | 7.7 | |
| OT/PT assistant | 2 | 7.7 | |
| Speech language pathologist | 2 | 7.7 | |
| Dietician | 1 | 3.9 | |
| Massage therapist | 1 | 3.9 | |
| Pharmacist | 1 | 3.9 | |
| Registered nurse | 1 | 3.9 | |
| Hours of work per week |  |  | |
| 10-20 | 1 | 3.9 | |
| 20-30 | 4 | 15.4 | |
| 30-40 | 17 | 65.4 | |
| 40-50 | 2 | 7.7 | |
| 50+ | 2 | 7.7 | |
| Hours of virtual care per week |  |  | |
| 0-10 | 19 | 73.1 | |
| 10-20 | 3 | 11.5 | |
| 50+ | 4 | 15.4 | |
| Out-of-hours care^a^ | 4 | 15.4 | |
| Work setting |  |  | |
| Inpatient | 16 | 61.5 | |
| Outpatient | 6 | 23.1 | |
| Mixed inpatient and outpatient | 4 | 15.4 | |
| Rehabilitation specialty area^b^ |  |  | |
| Musculoskeletal | 13 | 50.0 | |
| Neurorehabilitation | 8 | 30.8 | |
| Cancer rehabilitation | 6 | 23.1 | |
| Pain | 6 | 23.1 | |
| Trauma | 6 | 23.1 | |
| General physiatry | 3 | 11.5 | |
| Geriatrics | 3 | 11.5 | |
| Psychosocial and mental health | 2 | 7.7 | |
| Spinal rehabilitation | 2 | 7.7 | |
| Complex continuing care | 1 | 3.4 | |
| Amputee | 1 | 3.4 | |
| Burns | 1 | 3.4 | |
| Augmentative and alternative communication | 1 | 3.4 | |
| Pediatric rehabilitation | 1 | 3.4 | |
| Post-COVID | 1 | 3.4 | |
| Number of sessions attended (out of six) |  |  | |
| None^c^ (only baseline demographics) | 1 | 3.4 | |
| One | 2 | 7.7 | |
| Two | 3 | 11.5 | |
| Four | 5 | 19.2 | |
| Five | 5 | 19.2 | |
| Six | 10 | 38.5 | |

*Note.* *N* = 26; *n* = 20 participated in interviews.

^a^Reflects the number and percentage of participants answering “yes” to this question.

^b^Does not add up to *N* = 26 or 100% as participants were able to choose more than one option.

^c^Only baseline demographics and measures collected for the trial.
