# Supplementary material for: Specialist Rehabilitation Providers’ Experiences With an Online Self-Compassion Course: Reflexive Thematic Analysis
Source: JMIR Rehabil Assist Technol. 2026 Jul 15;13:e81706. doi: 10.2196/81706 (PMC13372217; doi:10.2196/81706)
Supplement: Multimedia Appendix 4 [file rehab-v13-e81706-s004.docx]

**Appendix E**

*Supplemental Quotes*

| Theme | Quote |
| --- | --- |
| Theme 1: The nature of working in rehabilitation | I only recognized in the last couple years of my career, was a lot of abuse and a lot of dumping, and a lot of negative energy put onto you. So, people don’t realize, or sometimes my other colleagues don’t realize after four to six weeks of someone being so negative, there is an impact on you. When you don’t think about it clearly and if they are mean to you or if they have high expectations on you, you carry that while that patient’s there. And that’s four to six weeks of that. So, you can have a family member that’s very abusive or very in your face, that’s four to six weeks that you have to deal with that person and their family. A person in acute care, they’re in and out and off they go, but we have four to six weeks of that, and it can put a toll. – Woman, age 45 |
|  | I think we work with people and we get to see them because we’re with them. In-patient rehab, we’re with them daily for two or three weeks on our floor. And we see them through a range of experiences. Generally, in the beginning, when things are a lot harder for them, where they can’t move as easily. They might be in more pain. They might be scared of falling or scared of not regaining their independence. Or with their family members who are really stressed and burnt out and not sure how they’re going to support their loved ones. So, being there for our patients. Or even as they get through, later on in their journey, when they’re planning for going home, which can be scary for them as well. And maybe as they realise that things are going to be a bit different, at least in the beginning where they are going to need more help. And that can be challenging for them. – Woman, age 42  My physio partner was off, sick, and they didn’t replace that person for the longest time. So, for months, we were short. So, having had to take on more myself and not having the adequate support, I know it was really getting to me. And then I was having… I was feeling stressed and overwhelmed with what I was trying to do. And I was also trying to think about things and do physio kind of tasks or things that normally I wouldn’t do because there wasn’t a physio. And so, it affected. I wasn’t sleeping as well. So, really, it was affecting me, both personally, emotionally, physically. And so, I know at that time, I really didn’t have… I didn’t feel at my best. And so, I know I wasn’t necessarily at my best with the patients either. So, I did what I could. But, normally, I know it’s not the same kind of therapeutic rapport or relationship that I could normally have when I’m not in that kind of a situation where it was just the demands were just too much for an extended period of time… Because we have high turnover of patients. We have length of stay targets that we have to meet. Our patients are more complex. And there’s a lot involved in discharge planning, which primarily falls on us OTs. So, yes, it is stressful. – Woman, age 42  Yes. I work with a lot of people who are at the most stressful times in their life. And they’re in an environment that they don’t necessarily want to be in, and they probably don’t want to be there at all. And it’s my job to encourage them to do things that they need to do, like brush their teeth, brush their hair, even when they’re feeling anxious, even when they’re feeling depressed, to get them out of bed. And oftentimes there’s a little bit of trauma that comes out for them as well, and it’s stressful to hear it sometimes. Just to understand what other people are going through, that empathy kicks in, so there’s that stress. And I also work in a palliative care unit, and that is stressful, too, in a different way. Because you don’t know when you’re going to see that person again. And you’re not sure if what you’re doing is in their best interest, but you try, you do your best. – Woman, age 26  The piece that I struggle with a lot is when we’re sending someone home to a situation or sending someone through the system in a situation that just feels uncomfortable. So whether it’s someone that’s cognitively or physically impaired and is going to struggle when they leave, or maybe we’re fighting with a patient and a family because they don’t want to, I don’t know, leave the hospital while they’re waiting for long-term care. – Man, age 39 |
|  |  |
| Theme 2: Different perspectives on burnout in specialist rehabilitation | I think burnout is just when you are mentally exhausted, mentally exhausted, physically exhausted. I know it can happen when there’s a huge demand, like a lot of patients coming in, and you’re being pulled in many directions, and maybe I can’t spend a lot of time reviewing one patient for me to look into further, and things get overlooked and mistakes happen…I feel like it’s a physical exhaustion. You’re tired. And mental exhaustion…You’ve just been doing too much, and you haven’t had the time to just relax and unwind, sleep well, not worry about the next thing that you have to do…And we see the same things over and over again, someone fell, someone broke their hip, someone is admitted to the hospital because they’re deconditioned. We see it all the time as healthcare workers. And we’ve seen people recover as well, so sometimes maybe if someone complains about their situation, maybe we’ve heard it so many times that we just are immune to it. – Woman, age 45 |
|  | Burnout, to me, means, oof, okay. That’s more of a general feeling of what is the word? It’s emotionally drained. Feeling emotionally drained. I suppose there’s a physical aspect too where you might be feeling tired. Physically tired. And to me, it’s similar. Maybe it goes hand in hand with compassion fatigue. But it’s a bit different. It’s more of a general outlook. – Man, age 43 |
|  | Burnout means there’s a level of physical tiredness. Burnout means that physical tiredness persists even if you go to sleep. Burnout means you in your body feel paralyzed because it’s hopeless. You feel like all the energy you’re putting in, you’re not going to get the outcomes that you want. You feel stuck. Burnout means you’re overwhelmed. You start caring less and less about things, professionally or personally. And you’re going down a downward spiral that’s an exponential downward spiral. It’s all adding up. – Man, age 35 |
|  | Burnout, to me, is being both emotionally, physically, I want to say spiritually, like just out of steam, and not able to get through the day without interventions. So, like there was a time when I was burned out and I needed to take naps during the day. I just was emotionally exhausted and in the afternoon I would take two hours out of work, and then I would work later, because I needed that energy to keep going. So that’s one example. I have a really hard time disconnecting emotionally from work. So, even when I was burned out, I was always, I’ve never reached the point where I don’t care about anything. I would still worry about people and worry about work, and what would happen to them. But it is that feeling like, I can’t take care of all these things all at once. And a bit of desperation, like how am I going to get through this, who’s going to help me, is anything bad going to happen to me? Those kinds of things would come up when I was feeling burnt out. – Woman, age 50 |
|  | I’ve been going through a very rough personal period of years, multiple, multiple months, and it’s making it sometimes harder to be at work and focus, and the compassion burnout, those kinds of things are quite present for me. But it’s reminding that I can’t do this work if my cup is already too full, that kind of stuff. – Woman, age 34 |
|  | I think it comes from all of these different facets of your life, and they all start to collide into each other. A problem, challenge at home or a conflict with friends is going to impact how you are at work. It’s going to impact your desire to take care of yourself. If you’re too upset to go to the gym, or you just want to crash in bed when you get home because you’re so tired, it’s wicked that way that it also… It very easily sneaks past the other care strategies you may have in place. And because it’s slippery like that, you may not notice for some time. At some point, you may notice it…It just feels like such a heavy weight of exhaustion. If you aren’t aware of it, I think some of the signs I was talking about before, or just you’re going through the day in a bit of a haze, I don’t know, a bit complain-y, maybe more irritable, I find just trying to… I think when I think about it for myself, I think about, how can I do the bare minimum but make sure I’m still getting what people need done? So it becomes this way of short-handing your day or short-handing your work. – Man, age 39 |
|  | Compassion fatigue is step one towards full burnout. And I think [unclear] without ever losing their compassion. And maybe those people who burn out without ever losing their compassion, maybe not losing their compassion is what led to their burnout. Maybe compassion fatigue is that first step you need to stop and take a step back, if that makes sense. – Woman, age 40 |
|  | To me, burnout is a full-on, I've really pushed too far. I think with compassion fatigue, ideally, the way I visualize it is if I can recognize that's what it is and can I do something to stop. You’re in a yellow zone still. Mind you, I don't know what the green zone looks like anymore. But I'm living in a constant yellow. But even recognizing that you're there and trying to do things to keep you more towards the lighter, paler shades of yellow. Trying, striving to get back to green still and seeing that as still a realistic potential goal. Whereas to me, burnout is now we've gone full-on into, I really let it go too long and I did not…That's it. It's gone out of control now. That's it. I'm failing at being able to get through just the day. It's the struggle to get through every day. To me, that's when it's burnout. – Woman, age 53 |
|  | The fatigue for me would be, you have all this content, you have all this information, and you want to somehow help as the healthcare professional. But when you feel like your hands are tied, either because there’s so many competing priorities where you have to be compassionate or you want to be compassionate and kind at the same time, it’s frustrating because it’s overwhelming that you’re not able to meet what you would want to be able to meet. – Man, age 35 |
|  | And I see it in my colleagues who… They get angry. They're so tired of being so kind and caring and compassionate and loving and just wonderful healthcare providers. They're so tired of doing that for every single person that the next person who asks them for something, they're probably going to snap. Yes. That's how I define it. That's, to me, burnout, fatigue. You need to step back because that next person who asks for it doesn't deserve any less than you gave everyone else for the past 20 years. – Woman, age 40 |
|  | But, yes, I guess just, after a while, you start to… It starts to weigh you down, especially when you hear some of the stories. Because the patients, they open up to you much more than they would with a doctor or a therapist. The assistants, we see them day in, day out, for an hour-long treatment. And they share so much of their life experience. And you listen with compassion. But it does wear you down after a while. – Woman, age 34 |
|  | I’ve had students before, and they’ve burnt me out too. Where emotionally and physically not able to complete all my tasks at work, it’s making me work longer hours, longer ours means I’m tired, not working out, resorting to bad food. So, it’s just a pile up of that. Versus compassion fatigue is more like I’m really it’s not like, I’m physically fine, but I just mentally not there and emotionally not capable of providing and empathetic ear to patients. – Woman, age 45 |
| Theme 3: A new perspective – less self-criticism, more self-compassion | Self-compassion is tough, because you’re looking for answers. And the easiest blame is yourself. You start to analyze your choices and you think, well, if I had done this or done that, perhaps this wouldn’t have happened. You’re always looking for a cause. And it’s most because life is so unpredictable and we never have all the information or the complete backstory, it’s easy just to blame yourself. Self-compassion I find is harder. I find it much easier to be kinder to others than to myself, because self-compassion is very challenging. – Woman, age 49 |
|  | I think I’ve got the self-love thing down, it’s the compassion, and being kind to myself when I don’t say the right thing or I do something that needs someone else’s correction or something like that. It’s just recognizing, because sometimes I will use those negative self-talk things, like, oh, you’re so dumb for doing that, and, this is why you are where you were, blah, blah, blah. And then I know right away that those are harmful, so it’s actually not just recognizing that they’re harmful, but filling the space with something positive. – Woman, age 50 |
|  | And, as a caregiver, compassion means, yes, I acknowledge in the here and now that you might be in a difficult situation. You are in pain. I’m here to listen to you. I may not be always able to physically or emotionally help. But to be present to the emotions that are happening to another person or even to myself…I think I’ve observed where we’re on timeframes where we have to jump from one patient to the next patient. And so, sometimes I’ve observed my colleagues maybe, oh, your 30 minutes are up and I’ve got to go. And maybe that person needed just a few extra moments because they were struggling that day with the pain or the exercise or whatever it may be. And they just needed a little bit more of their attention in terms of listening and being empathetic and hearing…I think work is, even though I physically leave work, it’s still at my head. It’s still roaming around in my head. And so, when I don’t sleep well, sometimes I actually see my day go through in my dreams. And maybe some of my patients that have had an effect on me that day, whether they made me agitated or angry or happy. – Woman, age 54 |
|  | Compassion means to me approaching with a gentleness, and an acknowledgement of pain or suffering, with this understanding that it’s part of the human experience, and it’s normal, this idea of connection through suffering. - Woman, age 26 |
|  | I had one patient once who tried to kill himself, and he had tried to kill himself in a very terrible way. And every day was a struggle to get him to do stuff with me. But he would always do it eventually. He and I got along very well. And then one day I just wasn't getting anywhere with him. I said, why don't we walk down to the cafeteria, I'll buy you a coffee. And that's what we did. And it wasn't an official therapy. But it's what he needed at the time, was just to get off the unit, to go sit, just have a regular 20 minutes. That's how I try to approach treating my patients with compassion. What I have planned for the day doesn't always matter. It's more what they need in that time. – Woman, age 40 |
|  | And one of my famous things I say is simple, like, my amputee patients, I say I will try to understand how you experience, but I actually don’t know because I’m not the one who actually is going through the amputation. So, I will not sit and say I understand you, because I actually don’t. – Woman, age 59 |
|  | I use compassion in my own work by exhibiting kindness to my clients, building rapport with my clients. Compassion when I exhibit compassion to other people, that leaves them space to talk freely and openly about their experience, and help me better understand and be able to support them. I think it also allows them space to verbally process what they’re going through, if it’s a compassion filled environment. – Woman, age 30 |
|  | I know I have much less patience for my family. It definitely translates into your personal life. I don't know. I feel like I've kind of given up on myself, so I just don’t even think about wanting to help myself. – Woman, age 34 |
|  | But I do think, the way I’ll describe it, I’ve had difficulties with self-compassion because I grew up in a very domineering system. You’re either wrong or you’re wrong. You’re never right. And with that, I’ve internalized a lot of that. I think this course, with the intention to me specifically may have opened a very slight door of compassion, access. I’m very hopeful that I can continue to open that door, and then maybe one day really get a big experience of it. Or it’ll just trickle over time, and next thing you know, the door’s completely open one day, and you didn’t expect it…For me, I felt that the doors of self-compassion have… I know others deserve it. I don’t feel deserving. And I think this course is offering me, well, no, you can get some too. We all deserve it. You’re not special. We’re special in our own way, but we’re not special, that egotistical I don’t need help, I don’t need compassion, everyone else does, I’ll do it on my own, I don’t deserve. We all do. Everyone deserves it…Self-compassion is, for me, a newer concept because I am quite harsh on myself, a hypercritical view, very judgmental. And then one sentence that changed the game for me from the course was when we were talking about self-touch and putting a hand on your heart, it felt hocus-pocus, like, come on, guys, we’re just putting our hand on our chest, not a big deal…But then when they [unclear] even if you don’t have the ability to put your hand on your heart, you could still have the intention of turning towards with compassion. That, to me, was a huge game-changer that at any given time, if I’m brushing my teeth, I’m showing myself self-compassion because I’m doing it for a higher goal, which is my health. I’m eating a sandwich. I’m showing self-compassion. – Man, age 35 |
|  | What compassion means to me is caring about another person, or caring about yourself, and trying your best to help that person in a way that you can. Feeling that you want to help them, and then actually following through and helping them, if possible. – Woman, age 40 |
|  | Self-compassion means having compassion for yourself as an individual, as a person. This is something I really resonated with in the course and thought about it in a new way. It’s like just feeling. Just remembering that you are a person too and you have things you’re working on. And having patience for yourself like you would a good friend. That was one of the things that I don’t know if it was new, but it was just reviewing that and just really working on that. Recognizing it’s something I have to continue to work on from the course. – Man, age 43 |
|  | Compassion, it translates into being able to identify those thoughts and feelings and treating them more kindly and understanding where they’re coming from. I learned in the course the definition of compassion. But what spoke to me most was the idea of common humanity…And when the negativity bias becomes most pronounced and I feel this overwhelming sense of anxiety, as it builds, I take a greater of sense of peace. My peace can be restored when I remind myself that I’m not the only person navigating these feelings and thoughts. That common humanity piece is really important to me. It’s really important to me because it makes me feel less isolated. – Woman, age 49 |
| Theme 4: Growing recognition of the importance of compassion for oneself and others | Because I’m going through some health challenges. And so, I’ve been using the practices to help me cope with what I’m going through. Like some of those elements of like, this is hard. And the common humanity. And some of those… Or whether it be the soothing touch or feeling the soles of my feet. Or those different practices have helped as I’m trying to, yes, deal with my own health challenges. And then other times, of course, the busyness of the unit, yes, so it’s been very helpful…And I think learning to be kinder to myself and more aware of what I say to myself and kinder in what I say to myself. The course has helped with that a lot too. Because I hadn’t realized that I could be really hard on myself. – Woman, age 42 |
|  | I guess it gives you the reassurance that it’s okay to be kind to yourself, it’s okay to be… to think about yourself. Because sometimes when we were growing up, you always think about others, doing this more for others, not for you. So, yes, it’s okay sometimes to just step back and take care of yourself first, before other people. – Woman, age 53 |
|  | But that's what I heard, was, it's okay that you're not doing all the things. Basically, for me, my big take-home was, just tell yourself the same things that you will tell your friend when she's having a bad time. Do the same that you do for other people. Tell yourself it's okay. And that is huge. Because, again, I have not heard it put that way…It's usually, have the feelings and let them go. And I'm like, yes, I get that. That's meditation. That's fine. But it's also okay if I didn't get a chance to do that because today was so bad or I was so overwhelmed with other things. But that's okay because that's today and we'll figure it out. And then it's that permission and that grace that I would give to my patients, my friends, my family all the time. Because nobody can be expected to do everything and be everything all the time, which I tell them. – Woman, age 53 |
|  | I started writing about myself and personally, and it was like I was so scared. I’ve been so scared to engage with myself in that sort of medium and to that level, but it was… I’m so glad I took that opportunity and took that risk, and I don’t think I would have without it being in the course. It just showed me that I could write to myself, that I could talk about these things that I find so hard and that have been so hurtful. And in doing that, it takes some of the poison out of them. That was really good. – Man, age 39 |
|  | Maybe slightly, in a positive way. Again, just being able to utilize a few more tools that maybe I haven’t before. I feel like I'm definitely in a space of trying to learn and grow and always try to be better and be as self-reflective as I can. So, I'm in that space and so this just enhanced where I'm at. – Woman, age 44 |
|  | I use the strategies. I know we said a lot. But the hand on heart thing which I think [participant] said, hand on thigh, just like a proxy. I’ve been doing that. Like I’ve been doing that with my family too. Like my wife when we talk, just to remind myself to be grounded. And just the practice of doing the exercises I think just made me more attuned to my patients and just helped me remember to hold space for people and have more patience. Immediately, I just felt like my practice was shifting a little bit. – Man, age 43 |
|  | I’m thinking about self-compassion a lot more, like I brought it up to a client this morning when she was talking to me about soothing touch actually, how she uses that just kind of instinctively, she doesn’t know why she’s doing it, but she does it. I was planning to take a look through the workbook and see if maybe there was an exercise we could try next week because I know she’s somebody who already buys into it. – Woman, age 30 |
|  | Similarly, these tools of compassion, self-touch, loving, kind [inaudible], interspersed throughout the week will build that muscle during the training time so when it actually needs to be there, we’re more likely to be able to use it…But I think what changed for me in this course is maybe seeing the common humanity with other highly trained healthcare professionals also struggling, so it validated the experience. And I guess that’s what made me ready for it versus when I’ve come across on my own or through various courses. - Man, age 35 |
|  | I was not one who is very comfortable talking in front of the wider group, but I really did appreciate listening to others. And I found some of the other participants were so thoughtful and open and vulnerable and really shared both their struggles and how the practices were really meaningful to them. And that was inspiring in a way, to know other people are also struggling similarly. And to see how the practices were helpful to them. I think it was really… I found it valuable to hear from other people. – Woman, age 42 |
|  | I liked that it was not just one hospital and not just one area in one hospital. It's not just all the physios or all the dietitians or all the… It was all of us. And it was nice to hear that we all feel the same. And whether it's personal life, private life, or a combination of the both, we're all in the same boat. – Woman, age 40 |
|  | For example, with the strategy where we were supposed to identify the emotion and then feel it in the body and then breathe into it, I found it applied in a lot of different situations. So, even when I was biking home, I’m a little bit impatient and I like to bike a little faster, and sometimes in Toronto there’s bike traffic, so there is someone who is slower in front of me. So, just breathing into the impatience and letting it exist there and then also saying why don’t you just relax and bike home more slowly, enjoy your bike ride…But without trying to push away what I was feeling, I just let it exist and breathe into it, and I found that that was… There were a lot of strategies like that where I could just apply them as needed, without needing to make it a separate homework thing. – Woman, age 29 |
|  | Our hospital's not that big. I remember a year ago or so, a patient died and it was dramatic. We don't get a lot of deaths in rehab… My colleagues were holding her hand as she died. And in rehab we're not so trained for that. I've done palliative care in the past. I've done acute care. I'm maybe a little better equipped to deal with it emotionally, but some of my colleagues were not…And I think being able to sit down as a group after that happened, no management, just us, and talk about how we were feeling from everybody's perspectives… We had our doctor come in and talk to us, and she's absolutely lovely. And hearing her talk about how there was nothing that could be done and how she was affected by it was really good. No one led the meeting. It was informal. That I feel is the most important thing for us to prevent burnout of any kind, is being able to be like, oh, that sucked. And all of us felt it. And then we can all help each other get past it. – Woman, age 40 |
|  | I find it really energizing to… I work on an interprofessional team, so I’ll speak with a coworker that has the same client, that works with them through a different lens, to sort of discuss what I’m experiencing and see if they’re experiencing things similarly or differently. And then maybe take on some of their strategies or their approaches, or just having that moment to vent to someone who understands and maybe is exhibiting the same thing. – Woman, age 30 |
|  | I’ve learned to be more kind with self-compassionate touch, through the course, again, which I’m finding really helpful. I try to leave work at work. It’s easier said than done, but for some reason having that physical environment really helps. Just like not taking any work home with me, not answering or even looking at emails, don’t even think about looking at that kind of thing at home…But also, if there is anything that’s still on my mind, I try to let it out immediately once I leave that environment. Just talk in vague ideas of, oh, I had an issue today, and I feel like I could have done it better, and just leave it at that. Or have you ever had the situation where your client has this, this, and this, and you do this, this, and this, and it doesn’t work?...And seeking comfort from colleagues who have similar experience, that is another way that I cope. And then also just trying to make sure that my home is a safe space. – Woman, age 26 |
|  | I think I often am using compassion with my clients. But I do find that professionally I don’t take on as much of a, I guess, direct empathy role. I have a little bit of a professional barrier, so I find it easier to almost provide the care and care part of it, rather than getting bogged down in the empathy and feeling what my clients are feeling, because they are going through often things that are really quite dramatic. – Woman, age 29 |
| Theme 5: Challenges engaging with the SCHC course | Many people I know are charting during their lunch hours, anyway. It’s not like that time that they have is off time. They’re actually still on the whole time. Many others are staying a little bit later after work. Throw on a wonderful opportunity, with this kindness, self-compassion, but then doing it at a time that’s not congruent with what we’re trying to teach, could be explored further…I think the benefit of doing it online is I got access to so many different healthcare facilities. I don't know if you noticed, there was a certain [workplace 1] crowd, there was a [workplace 2] crowd. There was different crowds. And sometimes when you’re sharing in an environment where people know you, you may or may not be as open with what you’re sharing because you see them every day. – Man, age 35 |
|  | I typically don't have a lunch hour. So, I just feel like to power down and go to a mindfulness for an hour, then power up right away, I think that's probably hard for people. - Woman, age 49 |
|  | I didn’t attend all the courses. I think I only attended two and then I really had to back out because it was causing a lot more stress in terms of my workload, and I sat there more anxious that anything, so it was setting my afternoons off…So you’re in the environment that you’re working in, so it’s really hard to disengage some of the things that you’re trying to heal yourself with, because you’re in that environment. The lunch time thing, yes, though it sounds really good, but sometimes clinicians like myself are working through their lunch just to cope for the day, just to stay on top of everything. – Woman, age 45 |
|  | And just also that it was over Zoom. I’m just not very… Well, I’m getting better at talking over Zoom. But it’s just not… When you can’t make that. Although, I struggle with eye contact to begin with. But when you’re not surrounded by people, physically, I don’t know. It’s just you can’t pick up on their clues and their body clues. And I don’t know. It’s just not a very comfortable environment to talk about your emotions or talk about your feelings. It just doesn’t feel right. – Woman, age 34 |
|  | I really liked it being online. I could still do it from work, I didn’t have to commute anywhere. I could be here, and also be at a course that’s working for me. It also meant that as the instructors have iterated, that if I needed to be more closed, I could actually be closed. I could turn off my computer camera, and I could sit here and listen, but I didn’t have to worry about how I was being perceived. I didn’t have to look engaged, I could just give what I had, and not worry about other people judging me for it. I feel like a lot of people in that class were able to take advantage of that…I think if we had specific examples that we could work with from a rehab perspective, saying, for example, your session with your client didn’t go well. Or maybe going even more specific, you are a physiotherapist, and your client said that you didn’t do this well, and now you’re thinking about it afterwards, and what sorts of thoughts might come up, and how would you address it. – Woman, age 26 |
|  | But seeing how we all are struggling in certain ways and have the same sort of pressures and challenges. But seeing then how other people could use the self-compassion and ideas and techniques is inspiring for me to do it in my own life. And then talking to colleagues here who are using it too. So, I don’t know how you could make it more tailored to rehab professionals. – Woman, age 42 |
|  | But when we look at learning, the forgetting curve is quite high. We’re already forgetting half the content today. It’s been a week. We want structured time zones where you reinforce the learning or the practice, and for that, there may be an opportunity for us to meet once every month, for example, for three months as a follow-up to solidify the learning. And, again, this is out of the scope of this pilot, but, generally, that’s how programs would work for optimal transferred learning. – Man, age 35 |
|  | I can understand how you want to reach new people and spread and broaden your reach. But just because you broaden your reach doesn’t mean you’ve done, that you’re changing anything. For those graduates, there needs to be a graduate program where they can jump back on and start reapplying. And so, that’s what I would do. I would make sure that you have a plan for a graduate program. – Woman, age 49 |
| Theme 6: Challenges to sustaining self-compassion in specialist rehabilitation | I would say most people who are struggling physically are also struggling emotionally. I used to work on the stroke floor of my hospital. And I found often with that type of illness injury, it was harder to get a read on really how much someone was struggling, especially if their speech was impacted. It was a little bit harder to connect with people because you couldn't quite tell all the time how much emotionally this has impacted. And you always assume that because it's a devastating illness injury, that it's been devastating to them mentally as well. – Woman, age 40 |
|  | And then you tell the doctor or a therapist what the patient disclosed with you. But the patient is not willing to take any further steps, so they’re just putting so much on you. But just not willing to talk to a professional who could actually help them. That’s frustrating for me. I feel like I get that a lot. – Woman, age 34 |
|  | Sometimes with patients, if they’re… I’m happy to work with anybody, as long as they’re trying their best. But if there’s a consistent message or behaviour that they’re not really trying their best or they’re really just passively allowing me to do all of the work. Again, putting me at risk and putting my energy of not value, then to me that’s where I get more compassion fatigue. – Woman, age 45 |
|  | When a client is injured, your pain obviously impacts your emotional experience, they aren’t two separate things. You often find emotional issues that maybe were pre-existing before the injury are exacerbated when you’re dealing with pain, because that’s taking up some of your tolerance to manage with daily stressors and things like that. It’s like old emotional baggage comes back up basically when you’re injured. – Woman, age 30 |
|  |  |
|  | That stress of having so much to do. Not feeling like you’re doing enough or good enough or well enough. It makes it hard sometimes to be present and to be compassionate and to show up in a way that you would want to sometimes for our patients. Because you just don’t have the resources, like the inner resources, because you’re spread so thin. – Woman, age 42 |
|  | And so, I want to be able to provide more for clients, but then there are these limitations on how much time we’re able to spend with them and how big the case load should be. And so, I think that some of the system constraints are what gives me the most empathy fatigue, if that makes sense. – Woman, age 29 |
|  | Some days are stressful because I’m asked to see four patients at a time. And there’s no helping around. Or my patient isn’t doing well and there’s three patients in the gym that I have to leave unattended because… Just the nonsense that goes on around here sometimes is frustrating beyond.  But I don’t know. How do I deal with stress? You just get through the day. You don’t have time to sit down and breathe. Or be compassionate. I feel like it could be in, discussed in the course. There is no time or no place around here to sit down and soothe yourself. We see patients back to back the entire day. The only time to sit down is lunchtime. But I don’t know. You just get through the day. – Woman, age 34 |
|  | Because I think the staffing is the issue. You might not know, but [unit] has the lowest occupational therapy staffing level. And, so, I’m struggling with taking care of a lot of patients. I often-times feel like I don’t give enough attention to them. So, that’s my issue. It’s more about I’m feeling guilty if I don’t give enough attention to them, and I know, if I have the time, I can make a lot of difference. But I just cannot. And after all these years I do make sure I don’t spend way too much time here. I know I can’t sustain that. – Woman, age 59 |
